# Explaining Physical Activity and Self-Rated Health Through Motivation and Perceived Service Quality: A Structural Equation Model

**DOI:** 10.3390/healthcare14040478

**Published:** 2026-02-13

**Authors:** Vojko Vučković, Klemen Širok, Marta Bon

**Affiliations:** 1 Faculty of Sport, University of Ljubljana, 1000 Ljubljana, Slovenia; marta.bon@fsp.uni-lj.si; 2 Faculty of Health Sciences, University of Primorska, 6310 Izola, Slovenia; klemen.sirok@fvz.upr.si

**Keywords:** service quality, exercise motivation, physical activity, self-rated health, self-determination theory, SEM, health promotion, fitness centers

## Abstract

Background/Objectives: Understanding the determinants of physical activity (PA) and health outcomes requires integrating environmental and motivational perspectives. Grounded in Self-Determination Theory (SDT), this study tested a sequential model in which perceived sport infrastructure service quality enhances exercise motivation, which subsequently increases PA and leads to better self-rated health (SRT). Methods: A total of 546 recreational adult exercisers completed validated questionnaires assessing sport infrastructure service quality (SQAS), exercise motivation (MPAM-R), PA (IPAQ), and self-rated health. Structural equation modelling (SEM) was used to examine the hypothesised relationships among variables. Results: The proposed sequential model was supported. Perceived service quality positively predicted exercise motivation (β = 0.255, *p* < 0.001), motivation significantly predicted PA (β = 0.266, *p* < 0.001), and PA was positively associated with self-rated health (β = 0.115, *p* < 0.005). Model fit indices indicated a good and acceptable fit to the data (CFI = 0.947, TLI = 0.935, NFI = 0.914, GFI = 0.931, RMSEA = 0.072, SRMR = 0.067, χ^2^/df = 3.85). Conclusions: The findings underscore the importance of high-quality exercise infrastructure as a key environmental factor that supports motivational engagement and promotes healthier behaviour patterns. Interventions aimed at increasing PA and improving perceived health should address both environmental quality and motivational processes.

## 1. Introduction

PA is a cornerstone of public health, clearly linked to numerous positive health outcomes, including improved self-rated health [1,2]. Regular PA plays an important role in preventing chronic diseases, alleviating symptoms of depression, stress, and anxiety, and promoting overall well-being. However, maintaining participation in PA remains a significant challenge for many individuals. This difficulty often arises from a complex interplay of psychological and environmental factors. Self-Determination Theory (SDT) provides a useful framework for examining how environmental factors influence exercise motivation, which in turn affects PA behaviour and health outcomes [3,4]. Ecological models of PA similarly suggest that supportive environmental features (e.g., accessible, high-quality facilities), alongside personal motivational factors, are key drivers of people’s activity levels [5].

One critical environmental factor in exercise settings is perceived service quality—the exerciser’s evaluation of facility conditions, equipment, staff, and overall atmosphere. In fitness and sport contexts, higher service quality has been linked to greater member satisfaction and loyalty [6,7]. A positive perception of the physical environment not only increases the likelihood of attendance but can also enhance individuals’ motivation and willingness to engage in exercise [8]. Dimensions such as facility quality, instructor competence, and staff performance contribute to a positive experience, which can lead to increased customer loyalty and retention [6]. Studies also indicate that multidimensional service quality affects consumer satisfaction and loyalty depending on the level of exercise involvement, suggesting that a higher quality experience can sustain involvement [9]. The expectation of service quality can also vary based on the demographic and motivational patterns of fitness centre users [10]. In other words, a well-maintained, user-friendly exercise environment may fulfil basic psychological needs, such as feeling comfortable, safe, and supported, thereby boosting intrinsic motivation to be active.

Exercise motivation is known to be a very strong predictor of PA levels and adherence [11,12]. One of the most commonly used theories to explain exercise motivation is SDT. SDT distinguishes between intrinsic and extrinsic types of motivation that regulate behaviour. Intrinsic motivation is defined as engaging in an activity because of its inherent satisfaction. When intrinsically motivated, a person experiences enjoyment, uses their skills, and feels personal accomplishment. In contrast, extrinsic motivation refers to engaging in an activity for instrumental reasons or to obtain an outcome separable from the activity itself, for instance, exercising because of appearance or social recognition. Research based on SDT consistently shows that intrinsic motivation, characterised by enjoyment and personal satisfaction, is closely linked to long-term exercise participation and higher PA [4,13]. Both intrinsic and extrinsic factors influence individuals’ decisions to start and maintain PA, affecting adherence and sustained effort [14,15]. The absence of motivation has been associated with failure to achieve PA goals and poorer health outcomes [15]. Low or insufficient motivation is widely recognised as a key factor contributing to irregular or discontinued PA, particularly among adults who face competing life demands or limited access to supportive environments. Therefore, understanding how to enhance people’s motivation is an important goal for interventions. SDT suggests that motivation quality improves when social and environmental conditions support a person’s needs for autonomy, competence, and relatedness. A high-quality exercise service (such as friendly staff, well-designed programmes, and clean, convenient facilities) may support these needs by making exercisers feel valued and competent, thereby fostering more self-determined forms of motivation.

It is well established that increased PA is strongly associated with better health outcomes. Regular PA helps prevent chronic disease, improves mental well-being, and contributes to higher SRH and quality of life. Recent studies also show that active Europeans report better SRH [16]. Adherence to PA guidelines is positively linked to self-efficacy, a proxy for health perception [17]. Furthermore, the positive relationship between PA and SRH is well documented. Higher levels of PA and cardiorespiratory fitness are consistently associated with better SRH across different age groups [2,18]. Health-related quality of life is a key benefit of PA, with strong evidence of a consistent positive association [1]. SRH can be influenced by PA through various mediating factors, such as emotional intelligence and psychosocial stress [19].

**Figure 1:**
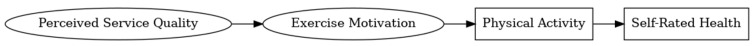
Hypothetical model.

While these relationships are individually supported, a comprehensive model that simultaneously examines the mediating roles of exercise motivation and PA in the relationship between service quality and SRH, particularly using a SEM approach, remains unexplored. Previous research has used SEM to analyse relationships between PA and quality of life [20], the mediating effect of psychological factors on PA [21], and the associations between motivation, satisfaction with life, and PA [13]. Other studies have also employed SEM to examine physical self-efficacy, psychological well-being, and organisational citizenship behaviour in relation to leisure-time PA [22]. However, a unified model encompassing service quality, motivation, PA, and SRH health within an SEM framework is essential to fully understand the mechanisms involved. While previous studies have demonstrated that both environmental conditions and individual motivation play important roles in influencing PA behaviour, they have largely examined these factors in isolation. Few studies have integrated these perspectives to explore how perceptions of service quality in exercise environments may influence motivational processes and, in turn, affect health-related outcomes. Moreover, there is limited empirical research applying a sequential, theory-based model to test these relationships in a diverse adult population. Based on these considerations, the present study aimed to test a sequential model linking perceived service quality → exercise motivation → physical activity → self-rated health in adult exercisers. We hypothesised that higher perceived service quality at exercise venues would strengthen individuals’ motivation to exercise, which would in turn increase their actual PA levels and ultimately lead to improved SRH. By examining this chain, our aim was to clarify how service quality and exercise motivation together influence health-related exercise behaviour. This makes our study unique. The research hypotheses and the hypothetical model (Figure 1) are presented below.

**H1.**  *Perceived service quality is positively associated with exercise motivation.*

**H2.**  *Exercise motivation is positively associated with PA.*

**H3.**  *PA is positively associated with SRH.*

**H4.**  *Perceived service quality has a positive indirect effect on SRH through a serial mediation of exercise motivation and PA*.

## 2. Materials and Methods

### 2.1. Study Design and Sample

We conducted a cross-sectional survey study among recreational adult exercisers in various community settings. To capture a diverse sample, participants were approached in multiple venues, including public parks, shopping malls, and outside fitness centres in Slovenia, a country with a high percentage (55.9%) of people who are sufficiently physically active. They were given iPads with short questionnaires. On the first page, they solved International Physical Activity Questionnaire (IPAQ) and SRH. Second page was for exercise motivation. And on the third page, we asked if they attend any health and fitness centre—if the answer was yes, they solved SQAS. This broad recruitment strategy was used to include people with different activity habits and background. A total of 867 adults initially agreed to participate. After excluding incomplete responses, the final sample consisted of 546 participants (those who answered all questions), approximately 63% of those recruited. Only complete cases were included in our analysis (listwise deletion). Of the 867 collected questionnaires, 546 contained complete data on all variables used in the model and were retained for analysis. Participants were aged 18 to 83, of which 278 women and 268 men. They ranged widely in age and fitness experience (covering young adults through older adults, casual exercisers to regular gym-goers, both men and women), reflecting the heterogeneity of the target population. All participants provided written informed consent, and the study protocol was approved by the Commission of the University of Primorska for Ethics in Human Subjects Research, number 4264-19-6/23.

### 2.2. Instruments

Data were collected using standardised, validated questionnaires administered on-site via tablets.

To assess service quality in health and fitness centres, we used the Service Quality Assessment Scale (SQAS), developed by Lam, Zhang, and Jensen [23]. This instrument measures six dimensions: staff (competence and friendliness), programmes (variety and quality of exercise offerings), locker rooms (cleanliness and comfort), physical infrastructure (orderliness and attractiveness of the environment), training areas (availability and functionality of equipment), and childcare (quality of ancillary services). The SQAS has proven to be a reliable and valid tool that enables systematic analysis of service quality and helps sports centres identify areas for improvement [23]. As childcare is not available in Slovenian fitness centres, this set of items was omitted, following the approach taken in the study by Yu et al. [24]. The SQAS scale was previously tested in Slovenian fitness centres in a pilot study [25] and showed good validity, with Cronbach’s alpha ranging from 0.885 to 0.950.

For the systematic assessment of reasons for engaging in PA, researchers use various questionnaires. One of the most commonly used instruments, but still relatively practical, based on SDT, is the Motives for Physical Activity Measure–Revised (MPAM-R). The MPAM-R assesses five main categories of motives: (1) health/fitness, (2) appearance, (3) competence/challenge, (4) social motives, and (5) enjoyment (Ryan et al., 1997) [26]. Items are rated on a seven-point Likert scale (1 = “not at all true for me” to 7 = “completely true for me”) [26]. A higher overall score reflects greater motivational drive to exercise. The original version consists of 30 items; however, a short form with 15 items (three per dimension) has also been developed, which retains the original structure and validity while being considerably more efficient [27,28]. For practical reasons, we used the shortened version. The short form has demonstrated reliability and validity across multiple studies and cultural contexts and is therefore suitable for both research and applied settings. This scale has demonstrated high validity, with Cronbach’s alpha ranging from 0.756 to 0.902, in adult exercise populations in a prior pilot study on a Slovenian sample [25].

We measured PA using the International Physical Activity Questionnaire—Short Form (IPAQ-SF). The IPAQ-SF captures self-reported frequency and duration of PA over the past 7 days across different intensity levels (walking, moderate, and vigorous activity) and yields a total PA score (e.g., MET-minutes per week). We categorised participants’ activity levels according to standard IPAQ scoring protocols and also used continuous scores for analysis. This instrument is widely used internationally and provides an estimate of habitual PA; it has been shown to be reliable and valid in a Slovenian sample [29].

SRH was assessed using a single-item global health question commonly employed in public health surveys: “How would you rate your overall health?” Participants responded on a Likert scale (e.g., 1 = poor, 5 = excellent). SRH is a subjective, single-item measure that captures an individual’s overall perception of their own health, including physical, mental, and social aspects, and serves as a strong indicator in research and health policy development. As it integrates multiple dimensions of health, SRH is considered a valuable, simple, and reliable tool for monitoring changes in health status and guiding public health interventions [30]. Although single-item indicators are less typical in SEM, SRH has consistently demonstrated strong construct validity and predictive utility in epidemiological and behavioural research [31,32], making it an appropriate and defensible outcome variable in structural models of health. For analysis, we treated this as an outcome variable reflecting the individual’s perceived health status, as demonstrated in previous studies [33].

### 2.3. Data Analysis

We tested the hypothesised sequential model using SEM with maximum likelihood estimation. In the model, perceived service quality was specified as an exogenous predictor, exercise motivation as a mediator between service quality and PA, PA as a mediator between motivation and health, and SRH as the final outcome. Composite scores (means) were calculated for each subdimension and used as observed indicators in a hierarchical path model. As the analysis relied on aggregated variables from validated and reliable instruments, an item-level confirmatory factor analysis (CFA) was not conducted. The SEM enabled us to examine direct and indirect paths simultaneously. Indirect effects were tested using bootstrap resampling (2000 samples) to obtain 95% confidence intervals. We assessed model fit with standard indices: Comparative Fit Index (CFI), Tucker–Lewis Index (TLI), Root Mean Square Error of Approximation (RMSEA), and Standardised Root Mean Square Residual (SRMR). A CFI or TLI above 0.90 and RMSEA or SRMR below 0.08 were considered indicative of acceptable model fit. Path coefficients were interpreted for significance (using *p* < 0.05 as the criterion). All analyses were conducted using the Python (version 3.13.) semopy library version(2.3.11), and we report standardised beta coefficients for the paths in the model.

## 3. Results

First, we measured reliability of MPAM-r short for our sample, which was shown to have good internal consistency, as we can see on Table 1.

As we can see on Table 2, SQAS scale for our sample has shown high internal consistency.

**Figure 2:**
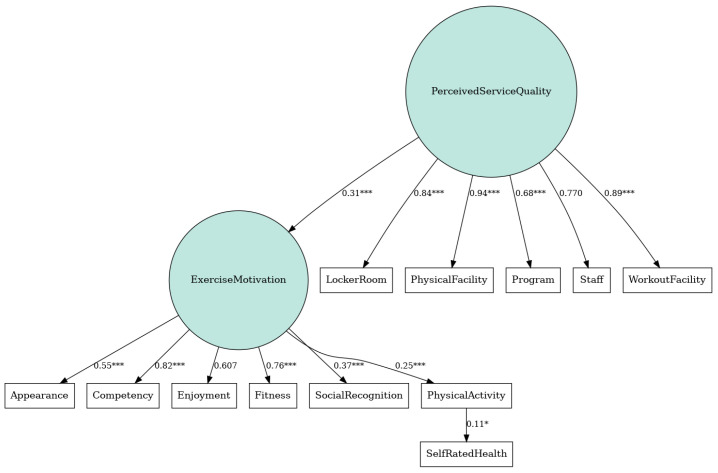
SEM, linking service quality, exercise motivation, PA and SRH. (* *p* < 0.05, ** *p* < 0.01, *** *p* < 0.001).

As we can see on the SEM on Figure 2, all hypothesised structural paths in the model were positive and statistically significant. Higher Perceived Service Quality was associated with greater Exercise Motivation (standardised β = 0.308, *p* < 0.001). In turn, Exercise Motivation positively predicted PA levels (β = 0.248, *p* < 0.001). Finally, PA had a significant positive relationship with SRH (β = 0.109, *p* = 0.01). Thus, participants who perceived better service quality at their fitness centres reported higher exercise motivation, which was linked to engaging in more PA, and those with higher activity levels, in turn, rated their health slightly better. All direct effects remained significant at the 95% confidence level or above. These results support a sequential pathway in which a supportive service environment influences motivational processes and behaviour, culminating in improved health perceptions.

The model demonstrated a good fit to the observed data, with fit indices falling within acceptable ranges: χ^2^ = 203.786, NFI = 0.914, TLI = 0.935, CFI = 0.947, GFI = 0.931, RMSEA = 0.072, and SRMR = 0.067. These values indicate that the proposed model adequately represents the relationships among the latent variables as we can see on Table 3.

The indirect effect of perceived service quality on self-rated health via exercise motivation and physical activity was statistically significant (standardised indirect effect = 0.008, 95% CI [0.002, 0.017]), indicating a significant serial mediation effect.

## 4. Discussion

This study examined a comprehensive sequential model clarifying the complex relationships between perceived service quality, exercise motivation, PA, and SRH among adult exercisers. Our SEM model showed an acceptable fit to the data, with all fit indices meeting established thresholds commonly recommended for structural equation modelling [34,35]. The findings, based on a robust SEM provide strong empirical support for the proposed theoretical framework and extend previous research by integrating these constructs within a unified model. Although previous studies have examined links between PA and quality of life [20], the role of psychological mediators in PA behaviour [21], and associations among motivation, life satisfaction, and exercise [13], few have tested an integrated model that simultaneously positions exercise motivation and PA as sequential mediators between perceived service quality and SRH. This study addresses that gap by providing a theoretically grounded and empirically tested pathway connecting environmental, psychological, behavioural, and health-related constructs.

Our model successfully integrates perceived service quality, an environmental factor, with internal psychological processes (exercise motivation) to predict behavioural outcomes (PA) and ultimate health perceptions (SRH). This is consistent with ecological models of health behaviour, which emphasise that individuals are shaped by their environment and that environmental factors can directly and indirectly influence health behaviours [36]. While previous research often examined these factors separately, our findings highlight the value of a holistic framework, demonstrating how supportive environmental features in exercise settings can foster motivation and subsequently promote health-enhancing behaviours [37,38]. This offers a stronger theoretical basis for understanding how external influences translate into internal drives for PA.

Our analysis confirmed Hypothesis 1, indicating a significant positive association between perceived service quality and exercise motivation. This finding enhances understanding of how external service attributes contribute to an individual’s internal motivation for PA. Consistent with existing literature, perceived service quality—including factors such as facility quality, instructor competence, and staff attitudes—significantly affects customer satisfaction and intentions in fitness contexts [6,10]. A high-quality exercise environment, characterised by well-maintained facilities, competent staff, and diverse programmes, appears to fulfil basic psychological needs, thereby fostering greater self-determined motivation [6,39]. This highlights that, beyond individual predispositions, the context in which PA occurs plays a pivotal role in motivating engagement.

Our analysis further supported Hypothesis 2, showing that exercise motivation is a significant positive predictor of PA levels. This finding aligns with a substantial body of research based on SDT, which posits that higher-quality motivation, particularly intrinsic motivation, is crucial for sustained exercise participation [4,40]. When individuals are genuinely motivated, driven by enjoyment and personal satisfaction rather than only external pressures, they are more likely to adhere to and maintain PA [41,42,43]. This highlights the need for interventions that not only promote PA but also foster autonomous forms of motivation, as these are often more predictive of long-term adherence [44,45].

In support of Hypothesis 3, we observed a significant positive relationship between PA and SRH, similar as other authors [2,18], contributing to individuals’ perceptions of their overall health [46]. While the effect of PA on SRH was modest in size, this is consistent with prior research using subjective health outcomes, which are influenced by a wide range of psychosocial and physiological factors. Given that the model tests a three-step mediation pathway and that the final outcome is a broad global health indicator, the observed indirect effect is necessarily small in magnitude. Nevertheless, the effect is statistically reliable and supports the proposed theoretical mechanism. Also, this is consistent with well-established epidemiological and clinical evidence identifying PA as a cornerstone of public health.

Importantly, our study provides robust evidence for Hypothesis 4, revealing a significant indirect effect of perceived service quality on SRH, serially mediated by exercise motivation and PA. This serial mediation pathway offers understanding of how environmental quality can indirectly influence health perceptions. It suggests that improving the perceived quality of exercise services enhances motivation, which then leads to greater PA and, ultimately, better SRH. This finding aligns with other SEM studies exploring the mediating roles of psychological factors and lifestyle behaviours on health outcomes [19,47]. The sequential nature of these relationships provides a valuable framework for designing comprehensive public health interventions aimed at improving SRH. For example, investments in facility quality, staff training, and programme design within community fitness centres could initiate a cascade of positive effects, leading to enhanced motivation, increased PA, and ultimately, better health outcomes for the population.

### Strengths and Practical Implications

Our study offers several notable strengths that enhance its scientific contribution and practical relevance. First, it addresses a critical gap in the existing literature by presenting a comprehensive, unified SEM that clarifies the sequential pathways from perceived service quality, through exercise motivation and PA, to SRH. This approach provides a more complex understanding of how environmental factors interact with psychological mediators and behavioural outcomes to ultimately influence an individual’s perception of their health. Integrating various theoretical constructs into a single model and exploring the interplay between environmental, psychological, and behavioural factors represents a significant theoretical contribution to health behaviour research, aligning with calls for more integrated models to better explain complex health behaviours [48,49,50,51]. Furthermore, the robust methodology, utilising a relatively large and diverse sample of 546 recreational adult exercisers recruited from various community settings, strengthens the generalisability of our findings beyond specific gym populations. The application of SEM with rigorous fit criteria allowed for the simultaneous assessment of multiple direct and indirect relationships, providing a statistically sound framework for evaluating our theoretical model. The use of validated and standardised instruments contributes to the validity of our results.

For health and fitness centres, the demonstrated positive association between perceived service quality and exercise motivation underscores the vital role of the exercise environment in promoting engagement. Previous research has consistently shown that customer expectations of service quality affect satisfaction and loyalty in fitness settings [6,10,39,52]. Our model confirms that investments in improving service quality—including well-maintained facilities, modern equipment, competent and supportive staff, and diverse programming options—can effectively enhance individuals’ intrinsic motivation to participate, resulting in greater adherence and sustained PA. Specific measures may include ongoing staff training in motivational communication, regular equipment maintenance and upgrades, and the implementation of responsive feedback mechanisms to address user concerns.

For public health interventions and policy makers, our results highlight that public health initiatives aimed at increasing PA should focus not only on promoting exercise itself but also on cultivating the underlying motivational factors. The serial mediation pathway shows that enhancing motivation is an important step in translating improved service quality into actual PA and, consequently, better SRH. Therefore, interventions could be designed to foster more self-determined forms of motivation, such as emphasising enjoyment, personal growth, and social connection, rather than relying solely on external pressures or incentives [4,40,41,44]. Policy makers could support community-based programmes that integrate high-quality, accessible facilities with motivational strategies, thereby addressing both environmental and psychological barriers to PA [42,43,45]. Last but not least, for individuals, such knowledge could encourage more informed choices about where and how to engage in PA, creating a more sustainable and enjoyable exercise that contributes positively to SRH [18,19,46,47]. By promoting awareness of these pathways, individuals can be better equipped to identify and cultivate conditions conducive to long-term PA adherence and improved SRH.

## 5. Limitations and Future Research

While this study offers significant contributions to understanding the relation between perceived service quality, exercise motivation, PA and SRH, several limitations warrant acknowledgment and provide avenues for future research. A primary limitation of the current study is its cross-sectional design. Although SEM enables the examination of complex relationships and pathways, it cannot establish definitive causal relationships or determine the temporal ordering of effects [53]. For example, while our model suggests that improved service quality leads to enhanced motivation and subsequent PA, a cross-sectional design cannot fully disentangle these dynamic processes. Future research would benefit from employing longitudinal designs to track changes over time and confirm the hypothesised causal directions. Such studies could include repeated measures of these constructs, providing stronger evidence for the proposed sequential model. Understanding motivational processes and PA over time, for instance, has increasingly been addressed through longitudinal studies [54,55]. Additionally, intervention studies designed to manipulate service quality or motivational strategies and observe their impact on PA and SRH would provide invaluable practical insight. Secondly, the exclusive use of self-reported measures for all key constructs introduces potential bias, including recall inaccuracies, social desirability, and overestimation—particularly in PA reporting [56]. Although we used well-validated instruments and ensured anonymous data collection to reduce such effects, self-reporting may still have led some participants to overstate their activity levels or perceived health, potentially inflating observed associations. Future studies should consider integrating objective tools, such as wearable activity trackers, to improve measurement accuracy and strengthen the validity of behavioural data. Also, because the SQAS was administered only to participants who reported using fitness facilities, the resulting structural missingness may limit generalizability and should be considered when interpreting the model’s scope. Finally, the sample consisted of recreational adult exercisers recruited from various community settings, which, while diverse, may limit the generalisability of these findings to other populations. The observed relationships might differ in clinical populations (such as individuals with chronic diseases or rehabilitation patients), specific age groups (such as adolescents or older adults with mobility limitations), or entirely sedentary individuals. Future research should strive to replicate this model across more diverse samples.

Despite these limitations, this study makes a significant contribution to understanding health behaviour by presenting a comprehensive SEM model that clarifies the sequential pathways from perceived service quality to SRH. The results support a holistic approach to public health strategies, highlighting the importance of creating supportive exercise environments that nurture intrinsic motivation to promote sustainable PA and, consequently, improve SRH.

## 6. Conclusions

Our findings confirm that people’s perceptions of the quality of their exercise environment are important, not only for satisfaction or attendance, but because they shape motivation to be active, which in turn influences actual activity levels and, ultimately, perceived health. This pathway from service quality, through motivation and behaviour, to SRH provides a valuable addition to current health promotion models. It highlights that encouraging PA is not only about telling people to move more, but also about creating environments that inspire them to do so. These insights are particularly relevant for public health strategies and facility managers aiming to increase participation and improve well-being across diverse populations.

## Figures and Tables

**Table 1:** Results of MPAM-r questionnaire reliability analysis.

MPAM-r	Cronbach’s Alpha	Items
Enjoyment	0.879	3
Fitness	0.824	3
Competency	0.842	3
Appearance	0.894	3
SocialRecognition	0.834	3

**Table 2:** Results of SQAS questionnaire reliability analysis.

SQAS	Cronbach’s Alpha	Items
Staff	0.942	6
Programme	0.918	6
Locker Room	0.935	5
Physical Facility	0.919	5
Workout Facility	0.938	6

**Table 3:** Model fit evaluation criteria and results.

Indices	Perfect Fit Limit	Acceptable Fit Limit	Scale Indices	Result
SRMR	≤0.08	≤0.1	0.07	Perfect
RMSEA	≤0.03	≤0.08	0.07	Acceptable
χ^2^/df	≤2	≤5	3.85	Acceptable
NFI	≥0.95	≥0.90	0.91	Acceptable
TLI	≥0.95	≥0.90	0.93	Acceptable
GFI	≥0.90	≥0.85	0.93	Perfect
CFI	≥0.95	≥0.90	0.95	Perfect

Abbreviations: SRMR (Standardised Root Mean Square Residual), RMSEA (Root Mean Square Error of Approximation), χ^2^/df (chi-square to degrees of freedom ratio), NFI (Normed Fit Index), TLI (Tucker–Lewis Index), GFI (Goodness-of-Fit Index), and CFI (Comparative Fit Index).

## Data Availability

Data is available upon request.

## Abbreviations

The following abbreviations are used in this manuscript:

CFI	Comparative Fit Index
GFI	Goodness-of-Fit Index
IPAQ	International Physical Activity Questionnaire
MPAM-R	Motives for Physical Activity Measure—Revised
NFI	Normed Fit Index
PA	Physical Activity
RMSEA	Root Mean Square Error of Approximation
SRH	Self-rated health
SDT	Self-Determination Theory
SEM	Structural Equation Modelling
SQAS	Service Quality Assessment Scale
SRMR	Standardised Root Mean Square Residual
TLI	Tucker–Lewis Index
